# High Proportions of GAP43 Positivity in the Cerebrospinal Fluids of Patients with Sporadic and Certain Types of Genetic Creutzfeldt-Jakob Diseases by Western Blot Analysis

**DOI:** 10.3390/ijms27041678

**Published:** 2026-02-09

**Authors:** Xiao-Xi Jia, Chao Hu, Jia-Feng Zeng, Bing Xu, Ju-Zheng Li, Ru-Han A, Dong-Lin Liang, Run-Dong Cao, Wei Zhou, Li-Ping Gao, Qi Shi, Cao Chen, Xiao-Ping Dong

**Affiliations:** National Key Laboratory of Intelligent Tracking and Forecasting for Infectious Disease, NHC Key Laboratory of Medical Virology and Viral Diseases, National Institute for Viral Disease Control and Prevention, Chinese Center for Disease Control and Prevention, Beijing 102206, China; sissi_chia@163.com (X.-X.J.); huchao@xwhosp.org (C.H.); a2017167510@163.com (J.-F.Z.); xubing1168@163.com (B.X.); 17379798390@163.com (J.-Z.L.); arh@ivdc.chinacdc.cn (R.-H.A.); liangdl@ivdc.chinacdc.cn (D.-L.L.); caord@ivdc.chinacdc.cn (R.-D.C.); zhouwei1970216@163.com (W.Z.); gaolp@ivdc.chinacdc.cn (L.-P.G.); shiqi@ivdc.chinacdc.cn (Q.S.)

**Keywords:** prion disease, GAP43, CSF, CJD, western blot, biomarker

## Abstract

Growth-associated protein-43 (GAP43) is a neuronal protein essential for synaptic function and plasticity, and its reduction has been observed in brains of prion diseases (PrDs) and rodent models. However, its status in the cerebrospinal fluid (CSF) of patients with PrDs remains unclear. CSF samples from 140 PrD cases, including 48 sCJD, 35 T188K-gCJD, 22 E200K-gCJD, 35 D178N-FFI, and 36 non-PrD controls, were analyzed for GAP43 by Western blot. The results were compared with 14-3-3 and calmodulin (CaM) detected by WB, and associated with clinical features. GAP43 positivity was significantly higher in sCJD (70.83%), T188K-gCJD (65.71%), and E200K-gCJD (72.73%) than in non-PrD controls (27.78%). The sensitivity and specificity of GAP43 (around 70–75%) were comparable to 14-3-3 and CaM, though inferior to RT-QuIC and total tau reported elsewhere. CSF GAP43 positivity correlated with sCJD-associated MRI changes, periodic sharp-wave complexes (PSWC) on EEG, and with 14-3-3 and CaM positivity. Our data here indicate the feasibility of usage of GAP43 by Western blot analysis as a diagnostic, at least as a screening, biomarker for sCJD and certain types of gPrDs.

## 1. Introduction

Human prion diseases (PrDs) are a group of fatal and transmissible neurodegenerative diseases, with sporadic Creutzfeldt-Jakob Disease (sCJD) accounting for about 85% of cases [1,2]. Approximately 5–15% of human PrDs are attributed to genetic mutations in PRNP, manifesting as genetic CJD (gCJD), fatal familial insomnia (FFI), and Gerstmann-Sträussler-Scheinker (GSS) syndrome. Definite acquired forms of PrDs are rare, such as iatrogenic CJD (iCJD), variant CJD (vCJD), and Kuru [3,4,5,6,7]. Although a definite diagnosis depends on neuropathological or PrP^Sc^ assays of postmortem or biopsy of brains, the real-time quaking-induced conversion (RT-QuIC) assay has revolutionized clinical diagnosis due to its high sensitivity and specificity using cerebrospinal fluid (CSF) or skin samples. Nevertheless, RT-QuIC is technically demanding and requires specialized equipment, limiting its availability in some clinical settings [8,9]. In parallel, exploration of the biomarkers in body fluid, particularly in CSF, has never ceased. A group of CSF proteins has shown diagnostic values, such as 14-3-3, tau, calmodulin (CaM), S100, Neurofilament light, β-synuclein, etc. [10,11,12,13]. Among them, CSF 14-3-3 by Western blot and total tau by ELISA are included in the diagnostic criteria for PrD in many countries.

Growth-associated protein-43 (GAP43) is a neuron-specific protein crucial for synaptic plasticity. Our earlier global brain transcriptomic assays of sCJD, FFI, and Alzheimer’s disease (AD) have identified a significant reduction in brain GAP43 levels. The proteomic study of the postmortem brains from sCJD, D178N-FFI, and G114V-gCJD patients also identified the downregulation of GAP43 [14,15,16]. More recently, using multiple methodologies such as Western blot, IFA, and IHC, we have observed that the brain levels of GAP43 are significantly decreased, whilst the phosphorylated form of GAP43 is elevated, in several scrapie-infected rodent models at the terminal stage, which shows close associations with neuron loss and PrP^Sc^ deposition during prion pathogenicity. However, little is known about CSF GAP43 alterations in PrD and its potential as a disease biomarker.

To address the possible changes of CSF GAP43 levels in PrD patients, the CSF samples from 140 different types of PrD cases and 36 non-PrD cases were selected in this study and subjected into GAP43 specific Western blot individually. Compared to the data of non-PrD (27.78%), the positive ratios of the cohorts of sCJD (70.83%), T188K- (65.71%), and E200K-gCJD (72.73%) were significantly higher, while that of D178N-FFI (20.00%) was remarkably low. CSF GAP43 positivity correlated well with the positivity of CSF 14-3-3 and CSF CaM in Western blots.

## 2. Results

### 2.1. Demographical and Clinical Characteristics

The main demographic and clinical features of all 176 cases were summarized in Appendix A. The median onset ages of sCJD, T188K-gCJD, E200K-gCJD, D178N-FFI, and non-CJD cases were 65, 62, 57, 53, and 58 years, respectively. Genotypic analysis revealed a predominance of Met/Met homozygote at codon 129 and Glu/Glu homozygote at codon 219 across all cohorts. Compared with the group of non-PrD, sCJD, and E200K-gCJD cases showed significantly higher raties of PSWC on EEG, MRI abnormalities, and CSF 14-3-3 positivity, while T188K-gCJD cases also exhibited higher rates of MRI abnormalities and CSF 14-3-3 positivity. No significant differences were observed for D178N-FFI versus non-PrD. Besides dementia, the frequency of other main sCJD-associated clinical symptoms was higher in all four PrD types than in controls.

### 2.2. GAP43 Positivity in CSF Across Various PrD Types

**Figure 1:**
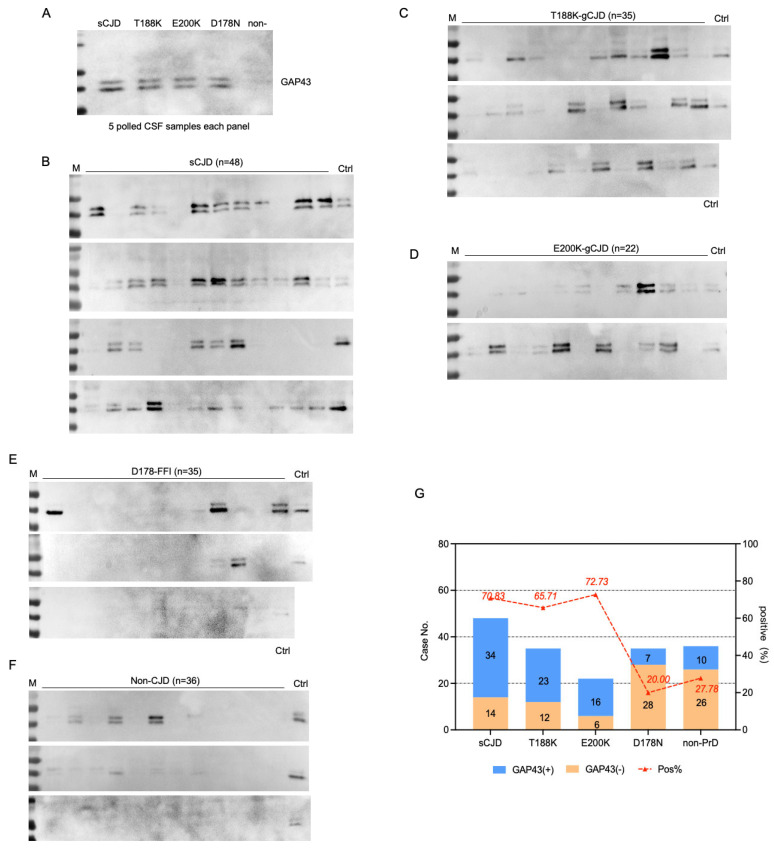
Determination of GAP43 in CSF specimens from the patients of sCJD, gPrD, and non-PrD using Western blots. (**A**) Blots of pooled CSF samples of various diseases, including sCJD, T188K–gCJD, and E200K–gCJD. D178N–FFI and non-PrD. Each pooled sample consists of an equal amount of CSF from five individual patients. (**B**) Blots of 48 CSF samples of sCJD cases. (**C**) Blots of 35 CSF samples of T188K–gCJD cases. (**D**) Blots of 22 CSF samples of E200K–gCJD cases. (**E**) Blots of 35 CSF samples of D178N–FFI cases. (**F**) Blots of 36 CSF samples of non-PrD cases. (**G**) The positivity of CSF GAP43 in the groups of sCJD, T188K–gCJD, E200K–gCJD, D178N–FFI and non–PrD. The case numbers of GAP43–positive and negative each group are shown in the column on right *Y*-axis and the positive percentage of GAP43 in each group is indicated at the top on the left *Y*-axis.

To address the CSF GAP43 status, Western blot analysis was performed on pooled and individual CSF samples. Each gel included a pooled sCJD-positive CSF sample as an internal normalization control. Two positive bands migrating at the position of 40 kDa were clearly detected in the CSF panels from sCJD and three other types of gPrDs, but were barely detectable in non-CJD samples (Figure 1A). Subsequently, equal amounts of CSF samples from 176 cases across different PrDs and non-CJD were individually blotted with anti-GAP43 antibody. Two specific bands with varying intensities were identified among different numbers of CSF specimens within each group (Figure 1B–F). The positive rates for CSF GAP43 were 70.83% (34/48) in sCJD, 65.71% (23/35) in T188K-gCJD, 72.73% (16/22) in E200K-gCJD, 20.02% (7/35) in D178N-FFI, 27.78% (10/36) in non-CJD, respectively (Figure 1G). Statistical analysis demonstrated significant differences among these groups (*p* < 0.001), with elevated GAP43 positivity in sCJD, T188K-gCJD, and E200K-gCJD compared to D178N-FFI and non-PrD (Table 1).

### 2.3. Correlation of CSF GAP43 Positivity with Clinical Features

The potential correlation of CSF GAP43 positivity was analyzed with key demographic, neurological, and clinical examination factors. The cases were categorized and counted based on CSF GAP43 positivity and negative status for different variables, and the statistical differences between positive and negative groups in various cohorts were calculated (Table 2). In the cohort of all PrDs, patients with GAP43 positivity displayed earlier onset ages than those with GAP43 negativity (*p* = 0.007). Patients who exhibited MRI abnormalities (*p* = 0.001), PSWC on EEG (*p* = 0.001), or mutism (*p* = 0.028) during their clinical courses had a higher proportion of positive CSF GAP43 results. Similarly, higher rates of CSF GAP43 positivity were observed in cases with recorded MRI abnormalities (*p* = 0.008) or PSWC on EEG (*p* = 0.011) within the cohort of all gPrDs. The remaining elements in these two cohorts did not reveal a statistical difference between GAP43-positive and negative groups. Moreover, no significant association was identified between CSF GAP43 positivity and selected clinical items within special PrD types (sCJD, T188K-gCJD, E200K-gCJD, D178N-FFI) as well as non-PrD.

### 2.4. Associations of CSF GAP43 with CSF 14-3-3 or CSF CaM

All 176 tested cases were addressed for Western blot data of CSF 14-3-3. To investigate the potential association of CSF GAP43 with 14-3-3, patients were categorized into GAP43-positive or negative groups according to their CSF 14-3-3 results. Statistical assays found that patients with CSF 14-3-3 positivity showed higher ratios of CSF GAP43 positivity in the cohorts of all PrDs (*p* = 0.008) and all gPrD (*p* = 0.018), whilst a lower ratio was observed in the cohort of non-PrD (*p* = 0.006) (Table 3). However, no statistically significant differences were identified among special PrD groups.

Out of the total 176 cases, Western blot results for CSF CaM were available for 152 cases. Analyses of the CSF GAP43 statuses in the groups of PrD showing CSF CaM positive and negative revealed higher GAP43 positivity ratios in CaM positive cases within the cohorts of all PrDs (*p* < 0.001), all gPrDs (*p* < 0.001) and T188K-gCJD (*p* < 0.001), but no statistical difference was observed in the other groups (Table 3).

### 2.5. Evaluation of the Diagnostic Performance of CSF GAP43

The diagnostic sensitivity and specificity of CSF GAP43 by Western blot for sCJD or gCJD (T188K and E200K) were comparatively evaluated alongside CSF 14-3-3 or CSF CaM. Based on the data from 48 sCJD and 36 non-PrD cases with both CSF GAP43 and CSF 14-3-3, the sensitivities of those two biomarkers were found to be 70.8% and 72.9%, while their specificities were determined as 72.2% and 75.0%, respectively. Similarly, using data from a cohort of 24 sCJD and 36 non-PrD patients with available information on both CSF GAP43 and CSF CaM, the sensitivities were calculated as 79.2% and 66.7%, whereas the specificities were estimated at approximately equal values of around 72.2% to 75.0%. In addition, among the group consisting of T188K- and E200K-gPrD cases (*n* = 57), Western blot analysis revealed that the sensitivities of GAP43, 14-3-3, and CaM were measured as 68.4%, 75.4%, and 80.7%, respectively, while their respective specificities were between 72.2% and 75.0% (Table 4).

**Figure 2:**
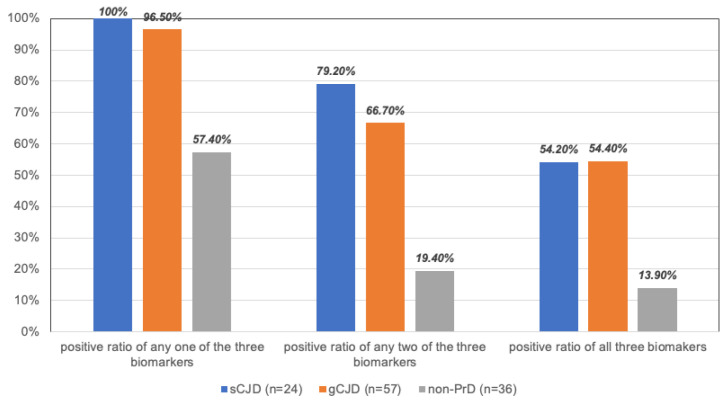
Positive percentages of CSF GAP43, 14-3-3, and CaM in the cohorts of sCJD, gCJD (T188K and E200K), and non-PrD. The positive ratios of any one of the three biomarkers (right), any two of the three biomarkers (middle), and all three biomarkers (left) are illustrated separately.

Further, the positive ratios of any one, two, and all three biomarkers in each group (sCJD, gCJD (T188K + E200K), and non-PrD) were separately calculated. As indicated in Figure 2, the positive rate of any one of the three CSF proteins by Western blot was 100% (24/24) in sCJD, 96.5% (55/57) in gCJD, and 57.4% (21/36) in non-PrD, estimating the specificity of 42.6%. The positive rates of any two of the three CSF markers in the cohorts of sCJD, gCJD, and non-PrD were 79.2% (19/24), 66.7% (38/24), and 19.40% (7/36), with a specificity estimate of 80.6%. Finally, in the same cohorts, a simultaneous positivity for all three CSF markers was observed in 52.2% (13/24) of sCJD, 52.4 (38/57) of gCJD, and 13.9% (5/36) of non-PrD, respectively, estimating the specificity of 86.1%.

## 3. Discussion

Over the past decade, RT-QuIC assays utilizing CSF and skin specimens have become the gold standard for the diagnosis of sCJD and some gPrDs, owing to their high sensitivity and specificity [17,18]. RT-QuIC has also shown reliable diagnostic value for certain types of gPrDs, such as E200K- and T188K-gCJD, but exhibits lower sensitivity for others like D178N-FFI [19]. However, the relatively complex procedures, specialized equipment, and technical requirements hinder the extensive utilization of RT-QuIC as a routine diagnostic tool in many clinical settings. In parallel, several CSF biomarkers, including 14-3-3, t-tau, and p-tau/tau, have been incorporated into diagnostic criteria for sCJD, while additional markers such as calmodulin and synaptic-related proteins (e.g., α-synuclein and β-synuclein) have been explored for their diagnostic potential [13,20,21,22]. Recently, aberrant changes in GAP43 have been reported to be associated with certain neurodegenerative diseases, prompting interest in its feasibility as a fluid biomarker for synaptic dysfunction and loss [23].

In this study, we evaluated CSF GAP43 as a diagnostic biomarker for PrDs using Western blot. GAP43 positivity was defined by rigorous densitometric quantification, with a strict cut-off value (mean + 2SD of non-PrD controls) and normalization to pooled positive controls. All blots were processed in parallel under standardized conditions to minimize technical variability and improve reproducibility. Around 70% of patients with sCJD, T188K-gCJD, and E200K-gCJD tested positive for CSF GAP43 signals, whereas positivity was observed in less than 30% of patients with D178N-FFI and non-PrD controls. These findings suggest that CSF GAP43 has potential utility as a screening biomarker, particularly for sCJD and certain types of gCJD subtypes.

Among gPrDs, T188K-gCJD, E200K-gCJD, and D178N-FFI represent the most prevalent subtypes in the Chinese population [19,24,25]. Coincidental with patterns reported for other CSF biomarkers (e.g., 14-3-3, total tau, CaM) as well as CSF RT-QuIC reactivity [12,26], CSF GAP43 positivity in T188K- and E200K-gCJD was comparable to that observed in sCJD. These similarities likely reflect shared neurodegenerative processes despite different etiological prion mutations. Furthermore, apart from abnormalities detected in CSF samples, T188K/E200K-gCJD exhibit similar neuropathological features as well as clinical manifestations and examination results (EEG and MRI) when compared to sCJD [24,25]. Conversely, D178N-FFI typically displays distinct neuropathological, clinical, EEG, and MRI characteristics compared with sCJD and T188K/E200K-gCJD, and correspondingly lower positivity rates for several CSF biomarkers, such as 14-3-3, total tau, CaM, and RT-QuIC [12,19,27]. Accordingly, CSF GAP43 detected by Western blot appears to be informative not only for sCJD but also for specific gPrDs that share pathogenetic features with sCJD.

GAP43 exhibits a high density at presynaptic termini and plays a key role in neuronal growth, actin modulation, synaptic plasticity, and vesicle transport, depending on its phosphorylation status [28]. Accumulating evidence suggests that synaptic dysfunction and loss represent early events in many neurological diseases, possibly occurring even before neuronal loss [29,30]. Our recent study has also identified down-regulated levels of brain GAP43 in several scrapie-infected experimental rodent models at the terminal stage, closely associated with neuronal loss and prion pathology [31]. In the present cohort, CSF GAP43 positivity correlated significantly with sCJD-associated MRI abnormalities, PSWC on EEG, and akinetic mutism, all of which are indicative of extensive brain injury and neuronal degeneration [32].

Consistent associations were also observed between CSF GAP43 and established CSF biomarkers detected by Western blot. Higher ratios of GAP43 positivity were identified in the groups positive for both 14-3-3 and CaM across all PrDs and within individual subtypes. Notably, patients of CaM positive reveal closer associations (higher OR values) with GAP43 positivity than those positive for 14-3-3 alone. While CSF 14-3-3 is an established biomarker included in the diagnostic criteria for sCJD [20], transient positivity has been reported in certain acute neurological diseases, e.g., encephalitis, ischemic stroke, paraneoplastic neuropathy, etc., likely reflecting rapid neuronal damage. Frequently, observations of CSF CaM positivity have been reported in patients with sCJD and certain types of gPrDs, including T188K- and E200K-gCJD [11,12]. Given that the interaction between CaM and GAP43 is involved in the regulation of intracellular calcium flux [33], further exploration into the mechanisms underlying the release of these neuroproteins into CSF may provide additional insights into prion disease pathogenesis.

The diagnostic sensitivity and specificity of CSF GAP43, 14-3-3, and CaM detected by Western blots were evaluated for sCJD and gCJD (both T188K and E200K) in this cohort. All three biomarkers demonstrated comparable diagnostic performance, with specificity and sensitivity generally ranging from 70% to 80%. Although slight differences in sensitivity were observed, these results support the potential role of GAP43 as a screening biomarker rather than a standalone diagnostic tool. Importantly, CSF GAP43 was positive in a subset of PrD patients who were negative for 14-3-3 or CaM, suggesting that its inclusion may improve overall diagnostic coverage within a multimarker strategy. Given that lumber puncture is typically performed only once during the clinical courses of suspected PrD, parallel assessment of GAP43, 14-3-3, and CaM by Western blot may help prioritize samples for further confirmatory testing, e.g., RT-QuIC. Combined positivity of two or three biomarkers significantly enhanced diagnostic specificity, achieving over 80.60% when two biomarkers were positive and exceeding 86.10% when all three biomarkers were positive concurrently.

Although CSF GAP43 shows lower diagnostic sensitivity than RT-QuIC or total tau, it provides complementary biological information by reflecting synaptic dysfunction, a core pathological process in prion diseases. GAP43 demonstrates consistently high positivity in sCJD and major gCJD subtypes and correlates with MRI, EEG abnormalities, and established CSF biomarkers. Therefore, GAP43 is best positioned as a supportive or screening biomarker rather than a replacement for existing gold-standard assays.

Western blotting was chosen because it remains widely accessible in prion surveillance laboratories and allows direct visualization of protein size and specificity, thereby reducing the risk of non-specific signal interference in CSF analyses. In addition, validated commercial ELISA kits for CSF GAP43 with standardized cut-off values are currently limited. Importantly, Western blotting has a higher detection limit than an optimized ELISA and may fail to detect low-abundance GAP43 in some CSF samples. Therefore, the Western blot–based positivity rates in this study should be interpreted as a conservative estimate, and a negative Western blot result should not be considered sufficient to exclude prion disease. We anticipate that a validated quantitative ELISA with standardized cut-offs could improve analytical sensitivity and potentially increase detection rates near the Western blot threshold.

This study has several limitations that should be acknowledged. First, GAP43 detection relied on Western blotting, which is inherently semi-quantitative and less sensitive than RT-QuIC or ELISA-based assays; This limitation may increase false-negative classifications and thereby constrain the negative predictive value of GAP43 when assessed by Western blotting alone. Second, although normalization procedures and blinded evaluation were implemented, the absence of a universal loading control for CSF remains a technical challenge and may affect inter-laboratory comparability. Third, this study did not directly compare GAP43 with RT-QuIC in individual cases, nor did it evaluate GAP43 in serum or plasma, which could provide more accessible and less invasive options for screening, especially among genetic mutation carriers. These limitations highlight the need for future research utilizing more sensitive and quantitative techniques, larger and independent cohorts, direct comparison with established diagnostic standards, and exploration of blood-based detection and longitudinal changes.

In conclusion, our results demonstrate that CSF GAP43 detected by Western blot may serve as a supportive biomarker for sCJD and certain gCJD subtypes. Further validation and methodological improvements are necessary before clinical implementation.

## 4. Materials and Methods

### 4.1. Patients

A total of 140 PrD (48 sCJD, 35 T188K-gCJD, 22 E200K-gCJD, 35 D178N-FFI) and 36 non-PrD cases, neurological controls were enrolled. The non-PrD control group included patients with Alzheimer’s disease (n = 1), autoimmune encephalitis (n = 4), epilepsy (n = 1), hepatic encephalopathy (n = 2), dementia with Lewy bodies (n = 1), limbic encephalitis (n = 1), multiple system atrophy (n = 1), along with other undiagnosed neurological conditions. These conditions were selected to represent disorders that may clinically or diagnostically mimic prion diseases, thereby allowing assessment of the specificity of CSF GAP43 in differential diagnosis. All sporadic Creutzfeldt–Jakob disease (sCJD) cases were confirmed to be homozygous for methionine (MM) at codon 129 of the *PRNP* gene. Molecular subtyping based on protease-resistant PrP^Sc^ isoforms (Type 1 or Type 2) was not uniformly available and therefore was not applied in this study. All diagnoses and differential classifications were performed by the National Surveillance for Creutzfeldt–Jakob Disease (CNS-CJD) at the China CDC, according to the Chinese national health industry standard: Diagnosis for Creutzfeldt–Jakob Disease (WS/T 562—2017) [34]. This diagnostic guideline, issued by the National Health Commission of China, was developed with reference to internationally recognized surveillance criteria, including the WHO manual for surveillance of human transmissible spongiform encephalopathies, the UK National CJD surveillance criteria, and the U.S. CDC diagnostic framework, ensuring international comparability of case classification. Under the national surveillance framework, each suspected case is submitted with standardized clinical information and ancillary investigations (e.g., MRI and EEG findings, CSF routine biochemistry, and prion-related laboratory tests when available) and is centrally reviewed by the National Prion Disease Surveillance Committee within the CNS-CJD program (China CDC). According to the national criteria, “Definite” cases require neuropathological and/or biochemical confirmation of PrP^Sc^; however, no autopsy/biopsy-confirmed cases were available in the present cohort. Therefore, all included cases were categorized as “Probable” under the national diagnostic criteria and surveillance protocol.

### 4.2. CSF Samples

The staff in local hospitals with routine lumbar punctures obtained the CSF samples of these patients. All CSF specimens were devoid of any blood contamination. After being transferred to the central laboratory in China CDC, the CSF specimens were centrifuged at 2000 rpm for 1 min, aliquoted, and stored at −80 °C. The results of the routine CSF biochemistry of the enrolled patients were collected via the CNS-CJD information system from the local hospitals, which were in the normal ranges of cell count, glucose level, and total protein level.

### 4.3. Western Blot (WB)

CSF (20 μL per sample) was separated by 12% sodium dodecyl sulfate-polyacrylamide gel electrophoresis (SDS-PAGE) and electronically transferred to nitrocellulose membranes (Whatman, Buckinghamshire, PA, USA), using a semi-dry blotting system (Bio-Rad, Hercules, CA, USA). After blocking, membranes were separately incubated with anti-GAP43 monoclonal antibody (mAb) (1:1000; Immunoway, San Jose, CA, USA), anti-14-3-3 polyclonal antibody (1:1000; Santa Cruz Biological, Santa Cruz, CA, USA), or anti-CaM mAb (1:1000; Millipore, Burlington, MA, USA) at 4 °C overnight. Membranes were further incubated with horseradish peroxidase-conjugated goat-derived anti-mouse antibody (Jackson ImmunoResearch Labs, West Grove, PA, USA; 115–035–003; 1:2000) at RT for 2 h, and the blots were developed by an enhanced chemiluminescence system (ECL; PerkinElmer, Waltham, MA, USA; NEL103E001EA). The images were captured by the ChemiDoc™ XRS + System with Image Lab software 5.2.1 (Bio-Rad) and quantified using Image J software 1.52 (National Institutes of Health).

### 4.4. Quantification and Definition of Positivity

For all targets, a pooled sCJD-positive CSF was included on every blot for normalization, and results were quantified by densitometry. All membranes were processed in parallel using standardized procedures to ensure reproducibility. For each sample, a visible band at approximately 40 kDa was considered positive if its intensity exceeded twice the mean value observed in non-PrD controls (cut-off: mean + 2SD of controls). All results were independently reviewed by two investigators blinded to clinical data to minimize bias. This definition was applied to all subsequent analyses. Blots that did not meet image quality standards were repeated.

### 4.5. Statistical Analysis

Data were processed using GraphPad Prism 10.1.1 (270) and SPSS 26.0 statistics software. The descriptive data were expressed as median (range) for continuous variables and percent for categorical variables. Categorical variables were compared using the chi-squared test, and continuous variables were analyzed using the Mann–Whitney U-test, adjusted by the Shapiro–Wilk test for normality. 

## Figures and Tables

**Table 1:** CSF GAP43 positivity in the different types of PrDs and non-PrDs.

Category	Disease	Mutation	No.	Positive (%)	χ^2^	*p*-Value
**sCJD**	sCJD	/ ^1^	48	34 (70.83)		
**gPrDs**	gCJD	T188K ^1^	35	23 (65.71)	37.975	<0.001 *
		E200K ^1^	22	16 (72.73)		
	FFI	D178N ^2^	35	7 (20.00)		
**non−PrDs**	non-PrDs	None ^2^	36	10 (27.78)		

Abbreviations: CSF, cerebrospinal fluid; gPrDs, genetic prion diseases; Significant difference between the groups marked with the different number (^1^ vs. ^2^) but no significant difference between the groups marked with the same number (^1^ vs. ^1^, ^2^ vs. ^1^) * Pearson chi-square test.

**Table 2:** Relationship of clinical features and CFS GAP43 positivity in total and different types of PrDs and non-PrDs.

Clinical	All PrDs			sCJD			All gPrDs			T188K-gCJD			E200K-gCJD			D178N-FFI			non-PrDs		
	GAP43+ (n = 80)	GAP43− (n = 60)	*p*-Value	GAP43+ (n = 34)	GAP43− (n = 14)	*p*-Value	GAP43+ (n = 46)	GAP43− (n = 46)	*p*-Value	GAP43+ (n = 23)	GAP43− (n = 12)	*p*-Value	GAP43+ (n = 16)	GAP43− (n = 6)	*p*-Value	GAP43+ (n = 7)	GAP43− (n = 28)	*p*-Value	GAP43+ (n = 10)	GAP43− (n = 26)	*p*-Value
Gender (M/F)	42/38	27/33	0.380 ^a^	18/16	6/8	0.525 ^a^	24/22	21/25	0.532 ^a^	14/9	7/5	1.000 ^b^	7/9	1/5	0.351 ^b^	3/4	13/15	1.000 ^b^	5/5	16/10	0.709 ^b^
Median onset age (y) (range)	60 (34–87)	56 (24–78)	0.007 ^c^	66 (48–87)	61 (37–78)	0.188 ^c^	59 (34–85)	54 (24–76)	0.077 ^c^	62 (40–85)	60 (50–76)	0.986 ^c^	58 (44–70)	52 (42–68)	0.356 ^c^	53 (34–62)	53 (24–70)	0.951 ^c^	53(42–73)	61 (22–73)	0.214 ^c^
MRI abnormality no. (%)	65 (81.3)	33/59 (55.9)	0.001 ^a^	32 (94.1)	13 (92.9)	1.000 ^b^	33 (71.8)	20 (44.4)	0.008 ^b^	19 (82.6)	9 (75.0)	0.670 ^b^	14 (87.5)	5 (83.3)	1.000 ^b^	0 (0.0)	6/27 (22.2)	N/A	5 (50.0)	4 (15.4)	0.079 ^b^
PSWC in EEG no. (%)	41/72 (56.9)	13/52 (25.0)	0.001 ^a^	28 (82.4)	8 (57.1)	0.142 ^d^	13/38 (34.2)	4/37 (13.2)	0.011 ^a^	5 (21.7)	2 (16.7)	1.000 ^b^	8/11 (72.7)	3/5 (60.0)	1.000 ^b^	0/21 (0.0)	0/4 (0.0)	N/A	1 (10.0)	1 (3.8)	0.484 ^b^
Progressive dementia no. (%)	74 (92.5)	53 (88.3)	0.401 ^a^	32 (94.1)	14 (100.0)	N/A	42 (91.3)	39 (87.8)	0.335 ^a^	21 (91.3)	10 (83.3)	0.594 ^b^	15 (93.8)	6 (100.0)	N/A	6 (85.7)	23 (82.1)	1.000 ^b^	8 (80.0)	20 (76.9)	1.000 ^b^
Myoclonus no. (%)	59 (73.8)	39 (65.0)	0.264 ^a^	30 (88.2)	9 (64.3)	0.127 ^d^	29 (63.0)	30 (65.2)	0.828 ^a^	14 (60.9)	9 (75.0)	0.476 ^b^	12 (75.0)	4 (66.7)	1.000 ^b^	3 (42.9)	17 (60.7)	0.430 ^b^	3 (30.0)	7 (26.9)	1.000 ^b^
Visual or cerebellar disturbance no. (%)	49 (61.3)	34 (56.7)	0.585 ^a^	19 (55.9)	6 (42.9)	0.412 ^a^	30 (65.2)	28 (60.9)	0.666 ^a^	17 (73.9)	9 (75.0)	1.000 ^b^	12 (75.0)	4 (66.7)	1.000 ^b^	1(14.3)	15 (53.6)	0.096 ^b^	1 (10.0)	6 (23.1)	0.645 ^b^
Pyramidal or extrapyramidal dysfunction no. (%)	62 (77.5)	46 (76.7)	0.907 ^a^	26 (76.5)	11 (78.6)	1.000 ^d^	36 (78.3)	35 (76.1)	0.804 ^a^	18 (78.3)	11 (91.7)	0.640 ^b^	14 (87.5)	5 (83.3)	1.000 ^b^	4 (57.1)	19 (67.9)	0.670 ^b^	5 (50.0)	12 (46.2)	1.000 ^b^
Akinetic Mutism no. (%)	47 (58.8)	24 (40.0)	0.028 ^a^	23 (67.7)	8 (57.1)	0.719 ^d^	24 (52.2)	16 (34.8)	0.092 ^a^	13 (56.6)	6 (50.0)	0.736 ^b^	10 (62.5)	4 (66.7)	1.000 ^b^	1 (14.3)	6 (21.4)	1.000 ^b^	1 (10.0)	2 (7.7)	1.000 ^b^

^a^ Pearson chi−square test; ^b^ Fisher’s exact test. ^c^ Mann−Whitney *U*-test. ^d^ continuity-adjusted chi-square test. N/A: Not Applicable

**Table 3:** Relationship of CSF GAP43 and CSF 14-3-3/CaM by Western blot and in PrDs and non-PrDs.

Disease	Mutation		CSF	GAP43+ (%)	GAP43− (%)	*p*-Value	OR (95%CI)
All PrDs	/	**14-3-3**	+	60 (65.2)	32 (34.8)	0.008 ^a^	1.565 (1.085–2.258)
			−	20 (41.7)	28 (58.3)		
		**CaM**	+	55 (74.3)	19 (25.7)	<0.001 ^a^	9.263 (3.838–22.356)
			−	10 (23.8)	32 (76.2)		
sCJD	/	**14-3-3**	+	26 (74.3)	9 (25.7)	0.613 ^b^	1.207 (0.753–1.935)
			−	8 (61.5)	5 (35.5)		
		**CaM**	+	13 (81.3)	3 (18.7)	1.000 ^b^	1.444 (0.189–11.042)
			−	6 (75.0)	2 (25.0)		
All gPrDs	/	**14-3-3**	+	34 (59.6)	23 (40.4)	0.018 ^a^	1.740 (1.049–2.885)
			−	12 (34.3)	23 (68.8)		
		**CaM**	+	42 (72.4)	16 (27.6)	<0.001 ^a^	19.688 (5.980–64.820)
			−	4 (11.8)	30(88.2)		
gCJD	T188K	**14-3-3**	+	18 (69.2)	8 (30.8)	0.736 ^b^	1.246 (0.658–2.359)
			−	5 (55.6)	4 (44.4)		
		**CaM**	+	23 (85.2)	4 (14.8)	<0.001 ^a^	3.000 (1.348–6.678)
			−	0 (0.0)	8 (100.0)		
	E200K	**14-3-3**	+	14 (82.4)	3 (17.6)	0.100 ^b^	2.059 (0.688–6.159)
			−	2 (40.0)	3 (60.0)		
		**CaM**	+	15 (78.9)	4 (21.1)	0.169 ^b^	7.500 (0.534–105.279)
			−	1 (33.3)	2(66.7)		
FFI	D178N	**14-3-3**	+	2 (14.3)	12 (85.7)	0.676 ^b^	0.600 (0.135–2.673)
			−	5 (23.8)	16 (76.2)		
		**CaM**	+	4 (33.3)	8 (66.7)	0.200 ^b^	3.333 (0.605–18.371)
			−	3 (13.0)	20 (87.0)		
non-PrDs	/	**14-3-3**	+	6 (66.7)	3 (33.3)	0.006 ^b^	4.500 (1.630–12.425)
			−	4 (14.8)	23 (85.2)		
		**CaM**	+	5 (55.6)	4 (44.4)	0.079 ^b^	5.500 (1.073–28.198)
			−	5 (18.5)	22 (81.5)		

^a^ Pearson chi-square test; ^b^ Fisher’s exact test; /: Not within the study’s focus.

**Table 4:** Evaluation of the diagnostic performance of 14-3-3 and GAP43 for sCJD and non-PrDs.

		sCJD (n = 48)	non-PrDs (n = 36)	Sensitivity(%)	Specificity(%)	gCJD (n = 57)	non-PrDs (n = 36)	Sensitivity(%)	Specificity(%)
14-3-3	+	35	9	72.9	75.0	43	9	75.4	75.0
	−	13	27			14	27		
GAP43	+	34	10	70.8	72.2	39	10	68.4	72.2
	−	14	26			18	26		
		sCJD (n = 24)	non-PrDs (n = 36)	Sensitivity(%)	Specificity(%)	gCJD	non-PrDs (n = 36)	Sensitivity(%)	Specificity(%)
CaM	+	16	9	66.7	75.0	46	9	80.7	75.0
	−	8	27			11	27		
GAP43	+	19	10	79.2	72.2	39	10	68.4	72.2
	−	5	26			18	26		

## Data Availability

The datasets generated for this study are available on request to the corresponding author.

## Appendix A

**Table A1:** The demography and clinical characteristics of the patients with PrDs and non-PrDs.

Clinical Features	Total PrDs (n = 140)		sCJD (n = 48)		Total gPrD (n = 92)		T188K-gCJD (n = 35)		E200K-gCJD (n = 22)		D178N-FFI (n = 35)		non-PrD (n = 36)
		*p*-Value vs. non-PrD		*p*-Value vs. non-PrD		*p*-Value vs. non-PrD		*p*-Value vs. non-PrD		*p*-Value vs. non-PrD		*p*-Value vs. non-PrD	
Gender (M/F)	69/71	0.333 ^b^	24/24	0.449 ^b^	45/47	0.338 ^b^	21/14	0.886 ^b^	8/14	0.104 ^b^	16/19	0.287 ^b^	21/15
Median age at onset (range) (y)	57 (22–73)	0.560 ^a^	64 (37–87)	0.018 ^a^	57 (24–85)	0.583 ^a^	62 (40–85)	0.201 ^a^	57 (42–70)	0.665 ^a^	53 (24–70)	0.031 ^a^	58 (22–73)
Age at onset < 50 years no. (%)	26/140 (18.6)	0.222 ^b^	4/48 (8.3)	0.018 ^b^	22/92 (23.9)	0.650 ^b^	3/35 (12)	0.036 ^b^	5/22 (22.7)	0.670 ^b^	14/35 (40)	0.276 ^b^	10/36 (27.8)
Age at onset 50–70 years no. (%)	93/140 (66.4)	0.225 ^b^	29/48 (60.4)	0.655 ^b^	64/92 (69.6)	0.134 ^b^	26/35 (74.3)	0.099 ^b^	17/22 (77.3)	0.095 ^b^	21/35 (60)	0.705 ^b^	20/36 (55.6)
Age at onset > 70 years no. (%)	21/140 (15.0)	0.805 ^b^	15/48 (31.3)	0.127 ^b^	6/92 (6.5)	0.077 ^b^	6/35 (17.1)	0.957 ^b^	0/22 (0)	0.115 ^c^	0/35 (0)	0.015 ^c^	6/36 (16.7)
Codon 129 genotype Met-Met/Total (%)	140/140 (100.0)	N/A ^c^	48/48 (100.0)	N/A	92/92 (100.0)	N/A	35/35 (100.0)	N/A	22/22 (100.0)	N/A	35/35 (100.0)	N/A	36/36 (100.0)
Codon 219 genotype Glu-Glu/Total (%)	114/115 (99.1)	1.000 ^d^	48/48 (100)	N/A	66/67 (85.7)	1.000 ^d^	24/25 (96.0)	0.417 ^d^	19/19 (100.0)	N/A	23/23 (100.0)	N/A	35/35 (100.0)
PSWC in EEG (%)	54/124 (43.5)	0.001 ^b^	36/48 (70.5)	0.001 ^b^	18/76 (22.4)	0.019 ^b^	7/33 (21.2)	0.116 ^c^	11/16 (68.8)	0.001 ^c^	0/25 (0)	0.580 ^d^	2/36 (5.5)
MRI abnormal change/Total no. (%)	98/139 (70.5)	0.001 ^b^	45/48 (93.8)	0.001 ^b^	53/91 (58.2)	0.001 ^b^	28/35 (80.0)	0.001 ^b^	19/22 (86.4)	0.001 ^b^	6/35 (17.1)	0.417 ^b^	9/36 (25.0)
CSF 14-3-3 Positive/Total no. (%)	92/139 (66.2)	0.001 ^b^	35/48 (72.9)	0.001 ^b^	57/92 (62.0)	0.001 ^b^	26/35 (74.3)	0.001 ^b^	17/22 (77.3)	0.001 ^b^	14/35 (40.0)	0.177 ^b^	9/36 (25.0)
Progressive dementia/Total no. (%)	127/140 (90.7)	0.065 ^c^	46/48 (95.8)	0.029 ^c^	81/92 (88.0)	0.142 ^b^	31/35 (88.6)	0.225 ^b^	21/22 (95.5)	0.135 ^c^	29/35 (82.9)	0.591 ^b^	28/36 (77.8)
Myoclonus no. (%)	98/140 (70.0)	0.001 ^b^	39/48 (81.3)	0.001 ^b^	59/92 (64.1)	0.001 ^b^	23/35 (65.7)	0.001 ^b^	16/22 (72.7)	0.001 ^b^	20/35 (57.1)	0.012 ^b^	10/36 (27.8)
Visual or cerebellar disturbance no. (%)	83/140 (59.3)	0.001 ^b^	25/48 (52.1)	0.002^b^	58/92 (63.0)	0.001 ^b^	26/35 (74.3)	0.001 ^b^	16/22 (72.7)	0.001 ^b^	16/35 (45.7)	0.018 ^b^	7/36 (18.4)
Pyramidal or extrapyramidal dysfunction no. (%)	108/140 (77.1)	0.001 ^b^	37/48 (77.1)	0.005 ^b^	71/92 (77.2)	0.001 ^b^	29/35 (82.9)	0.002 ^b^	19/22 (86.4)	0.003 ^b^	23/35 (65.7)	0.116 ^b^	17/36 (47.2)
Akinetic Mutism no. (%)	71/140 (50.7)	0.001 ^b^	31/48 (64.4)	0.001 ^b^	40/92(43.5)	0.001 ^b^	19/35 (54.3)	0.284 ^c^	14/22 (63.7)	0.001 ^b^	7/35 (20.0)	0.284 ^c^	3/36 (8.3)

^a^ Mann-Whitney *U* test; ^b^ Pearson chi-square test; ^c^ continuity-adjusted chi-square test; ^d^ Fisher exact test; N/A: Not applicable.
